# Scorpionism at the human–environment interface: an eco-epidemiological synthesis and conceptual framework

**DOI:** 10.3389/fpubh.2026.1771779

**Published:** 2026-03-09

**Authors:** Ahmed Karmaoui, Denis Sereno

**Affiliations:** 1 Faculty of Science and Techniques (Health and Environment Research Team, Errachidia),Moulay Ismail University (UMI), Meknes, Morocco; 2 INTERTRYP, Univ Montpellier, Cirad, IRD, Montpellier, France; 3 GoInsEct: Global Infectiology and Entomology Research Group, Montpellier, France

**Keywords:** eco-epidemiology, envenomation, neglected conditions, scorpionism, socio-ecological systems, tropical health

## Abstract

**Introduction:**

Scorpion envenomation is a major but persistently under-recognized public health problem in tropical and subtropical regions. Its occurrence arises from complex interactions among climatic, ecological, socioeconomic, and health-system drivers. Despite sustained high incidence and expanding risk associated with climate and land-use change, these relationships remain insufficiently synthesized. Addressing this gap requires an integrative framework capable of capturing the multi-level determinants shaping scorpionism risk and outcomes.

**Methods:**

Drawing on Human Ecology Theory and socio-ecological systems thinking, we developed an integrated conceptual model to organize determinants of scorpion envenomation. The framework synthesizes evidence from environmental science, climate impacts, scorpion biology, venom characteristics, epidemiology, and clinical and public health interventions. More than thirty determinants were categorized into six components: (1) physical–climatic variables, (2) scorpion behavior and ecology, (3) venom composition and toxicity, (4) human exposure and envenomation risk, (5) health impacts and public health response, and (6) socioeconomic factors. The model is structured in two stages: a risk-and-vulnerability stage and a scorpionism-occurrence stage.

**Results:**

The resulting conceptual model provides a unified systems-based framework that integrates environmental, ecological, behavioral, socioeconomic, and health-system determinants of scorpion envenomation. It clarifies pathways linking upstream drivers to exposure, clinical outcomes, and public health responses, enabling interpretation of regional heterogeneity in incidence. The framework also identifies priority leverage points for intervention and offers a structured basis for empirical research, risk mapping, and early-warning development.

**Discussion:**

This work highlights the importance of adopting a socio-ecological systems perspective to better understand and address scorpionism. By integrating diverse determinants into a coherent analytical framework, the model serves as a hypothesis-generating and interpretive tool rather than a predictive model. The approach supports the development of targeted prevention and control strategies and reinforces the need to recognize scorpionism within the broader agenda of neglected tropical health conditions, particularly given its substantial burden and strong socio-ecological determinants.

## 1 Introduction

Scorpions possess venom stored in telson glands (1). Stings occur predominantly in tropical and subtropical regions (2), causing more than 1.2 million cases and thousands of deaths annually (3). Globally, approximately 2,612 scorpion species have been described (4), although only 46–50 are considered lethal to humans (5). Clinical severity depends on species and venom potency, with different taxa inducing varying degrees of envenomation (6–8).

Taxonomically, the families Buthidae, Hemiscorpiidae, and, to a lesser extent, Scorpionidae comprise nearly all species responsible for severe human envenomation. This taxonomic and functional diversity has major implications for venom potency, clinical manifestations, and geographic risk, underscoring the need for conceptual approaches capable of capturing interspecific variability and associated epidemiological patterns without focusing on individual species.

Arid and semi-arid regions of North Africa and the Middle East (MENA) report particularly high scorpionism incidence, especially where access to healthcare is limited (9, 10). Across Morocco, Algeria, and Tunisia, envenomation displays a marked seasonal peak during summer (11–16). Environmental factors, including temperature, humidity, rainfall, and sunshine, strongly modulate scorpion activity and sting frequency (17–20), while climate change is expected to further intensify these risks (21). In parallel, social vulnerability—characterized by illiteracy, rural residence, poverty, and agricultural occupations—further amplifies exposure (3, 22–26).

Although conceptual modeling is widely applied in ecological, environmental, and public-health research (27–32), scorpionism has rarely been examined through such systems-based perspectives, with the notable exception of a recent spatiotemporal Bayesian analysis (22). While scorpionism research is inherently multidisciplinary, spanning toxinology, ecology, epidemiology, clinical medicine, and the social determinants of health, these domains remain poorly integrated. This fragmentation limits the ability to synthesize evidence, anticipate risk under climate and land-use change, and design integrated prevention and control strategies.

Despite extensive research on scorpion biology, venom, epidemiology, and clinical management, knowledge remains fragmented across environmental, ecological, social, and health-system domains, limiting translation into predictive risk assessment, early-warning systems, and integrated prevention strategies. Here, we develop a two-stage, six-component, species-agnostic socio-ecological framework that integrates determinants across the exposure-to-outcome continuum and links them to intervention entry points. Beyond existing DPSIR and One Health approaches, this framework explicitly resolves the sequential transition from environmental drivers to human exposure and clinical burden, providing a basis for hypothesis testing, indicator selection, risk mapping, and evaluation of multi-sectoral interventions.

To operationalize this framework, we formalize an integrated conceptual model synthesizing climatic, ecological, venom-related, behavioral, socio-economic, and health-system determinants of scorpionism within a socio-ecological systems structure. By organizing these interactions into a risk and vulnerability stage and a scorpionism occurrence and response stage, the model provides a unified foundation for empirical modeling, vulnerability mapping, early-warning systems, and targeted intervention design in endemic regions.

## 2 Methods

### 2.1 Literature search strategy

We conducted a structured narrative synthesis of the scientific literature on scorpionism to identify determinants relevant to building a socio-ecological conceptual model. We did not aim for exhaustive retrieval; rather, we sought cross-disciplinary coverage to inform framework construction. To mitigate selection bias, we purposively sampled across toxinology, ecology, epidemiology, and health-systems domains and cross-checked determinants against reference lists of key reviews and regional surveillance syntheses. In addition, because the objective was conceptual integration rather than quantitative meta-analysis, we did not follow PRISMA guidelines. However, to enhance transparency, we document below the databases searched, search terms, inclusion/exclusion criteria, screening approach, and the process used to extract and map variables. Database searches were conducted from inception to December 2024, with the final search completed in December 2024.

#### 2.1.1 Databases and sources consulted

The following major scientific databases were searched:

- PubMed/MEDLINE
- Scopus
- Web of Science Core Collection

To complement peer-reviewed literature, additional targeted sources included:

- Reference lists of eligible articles
- WHO technical reports
- National surveillance bulletins from MENA and Latin America (when cited in the peer-reviewed literature)

No language restrictions were applied, although >95% of included literature was available in English or French.

#### 2.1.2 Search terms

Searches combined terms related to scorpion biology, ecology, epidemiology, public health, and socio-ecological drivers.

#### 2.1.3 Core search strings

- “scorpionism”
- “scorpion sting” OR “scorpion stings”
- “scorpion envenomation”
- “scorpion* AND epidemiology”
- “scorpion* AND ecology”
- “scorpion* AND climate”
- “scorpion venom” AND “toxicity”
- “risk factors” AND “scorpion*”
- “socioeconomic” AND “scorpion*”

#### 2.1.4 Inclusion criteria

Articles were retained if they met at least one of the following criteria:

1. Epidemiological studies reporting incidence, seasonality, clinical severity, hospitalization, or mortality of scorpion stings.
2. Ecological or biological studies describing scorpion species, distribution, activity cycles, habitat preferences, reproduction, or behavior.
3. Toxinological studies describing venom composition, venom plasticity, or mechanisms of toxicity.
4. Environmental and climatic studies associating sting incidence or scorpion abundance with temperature, humidity, rainfall, wind, vegetation, or land use.
5. Socio-economic studies linking scorpionism to social vulnerability, occupational exposure, poverty, housing conditions, or urbanization.
6. Public-health studies on antivenom availability, health-system response, prevention strategies, or awareness programs.
7. Conceptual modeling literature relevant to socio-ecological systems, health frameworks, or environmental health indicators.

All study designs were eligible: cross-sectional, cohort, case–control, ecological, descriptive, experimental, and review articles.

#### 2.1.5 Exclusion criteria

Studies were excluded if they:

- Focused exclusively on scorpions in captivity with no ecological or epidemiological relevance
- Provided purely taxonomic updates without behavioral, ecological, or health-related information
- Concerned venom purification without discussing toxicity or clinical relevance
- Were conference abstracts without accessible full text

#### 2.1.6 Study selection process

1. Initial screening was performed based on titles and abstracts to remove irrelevant records.
2. Full texts were reviewed for all remaining articles.
3. From eligible studies, relevant information were extracted and grouped into six components (Physical–Climatic, Behavior/Ecology, Venom, Human Exposure, Health Impacts/Response, Socio-Economic).
4. All included references appear in the main manuscript’s bibliography (*n* ≈ 150).

#### 2.1.7 Approach to variable extraction and model development

For each eligible article, the information was recorded:

- Variable(s) addressed (e.g., temperature, habitat fragmentation, age of victims, venom dose)
- Direction and nature of associations with scorpionism
- Ecological or socio-economic context
- Scale of analysis (local, regional, national)

The variables were then:

1. Aggregated across studies,
2. Categorized into the six components, and
3. Mapped into the conceptual framework (Risk/Vulnerability → Occurrence/Response).

**Figure 1:**
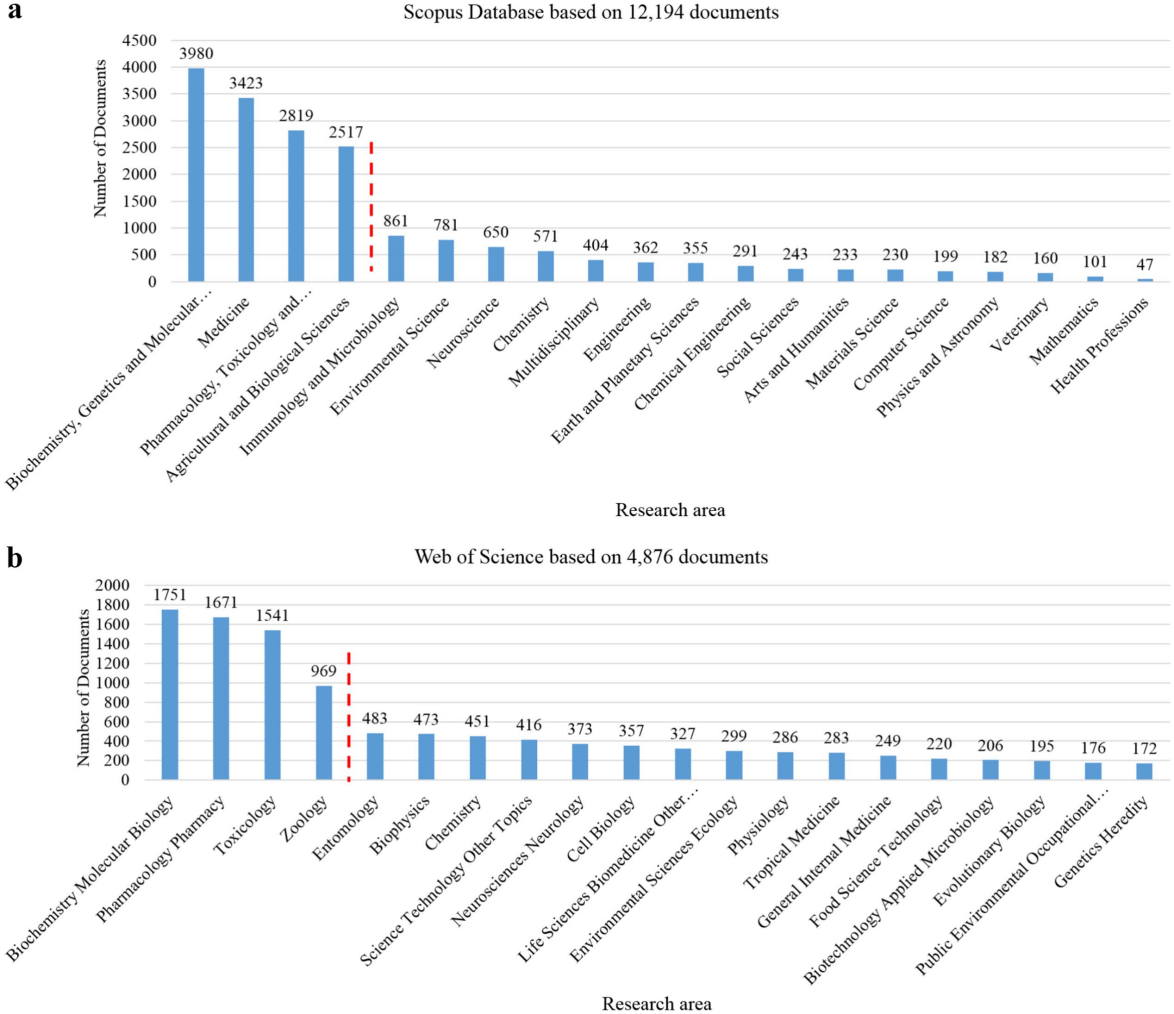
Integrated determinants of scorpionism risk and vulnerability (Stage 1). This figure represents the first stage of the conceptual model, focusing on the generation of risk and vulnerability to scorpionism. It illustrates how interacting physical and climatic variables (e.g., temperature, rainfall, humidity, vegetation, and soil characteristics) influence scorpion distribution, abundance, and activity. These environmental drivers interact with scorpion behavior and ecology, including activity cycles, habitat selection, and dispersal patterns, which together determine the probability of human–scorpion contact. Venom composition and toxicity add a biological dimension to risk, with venom dose and biochemical profiles varying across species and environmental contexts. Human exposure and envenomation risk emerge from the interaction between scorpion ecology and socio-economic conditions, such as rural residence, occupation, housing quality, and access to healthcare.

**Figure 2:**
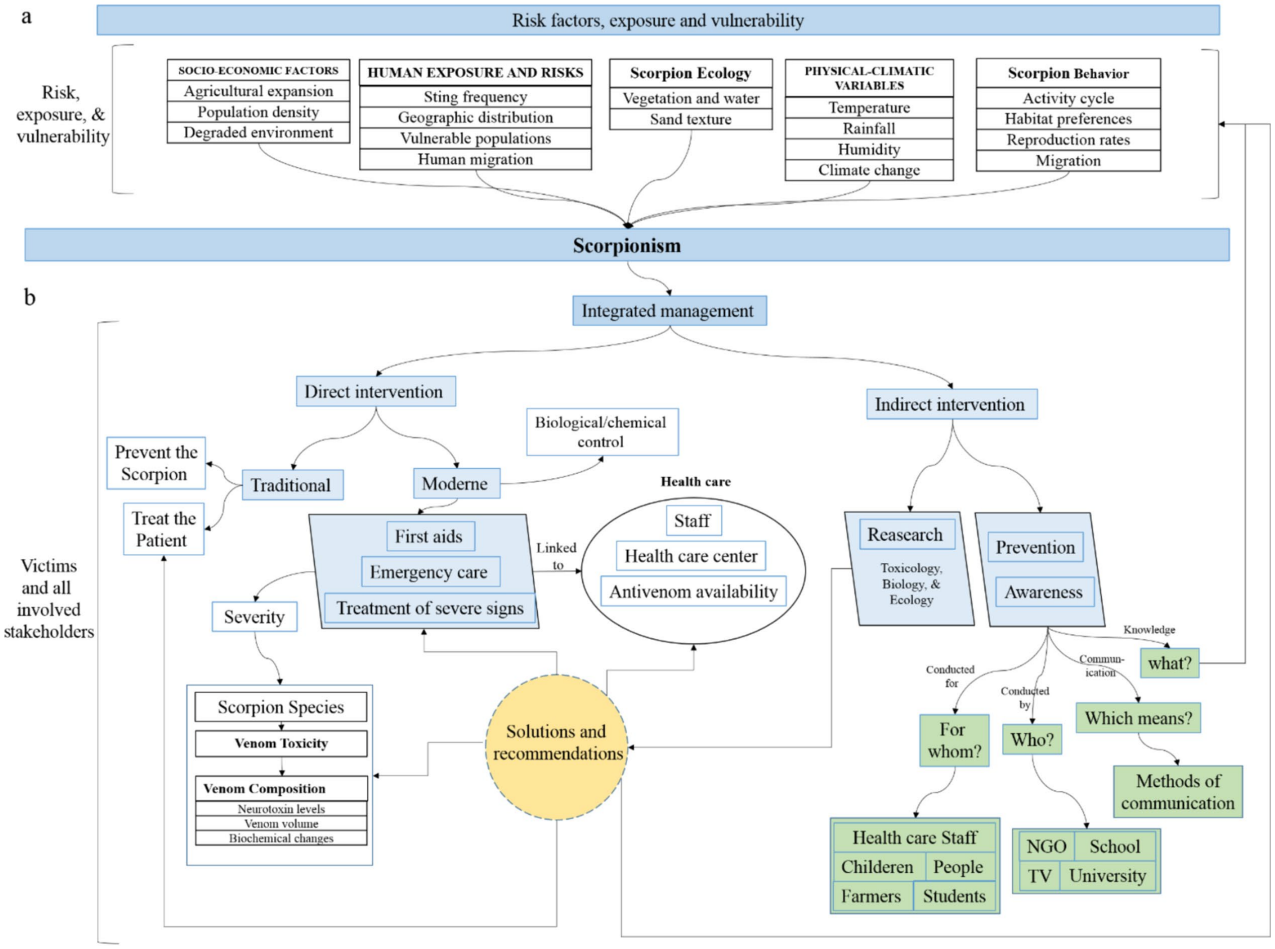
Integrated conceptual model of scorpionism: from risk and vulnerability to occurrence and intervention pathways. This figure presents the integrated conceptual model of scorpionism, combining risk generation, exposure, and response pathways within a unified socio-ecological framework. Panel **(a)** represents the risk, exposure, and vulnerability stage, illustrating how interacting physical–climatic variables (e.g., temperature, rainfall, humidity, climate change), scorpion ecology and behavior (activity cycles, habitat preferences, reproduction, migration), environmental characteristics (vegetation, water availability, substrate), and socio-economic factors (agricultural expansion, population density, environmental degradation) jointly shape human exposure and vulnerability. These upstream determinants converge to produce scorpionism occurrence, depicted at the interface between stages. Panel **(b)** illustrates the occurrence, management, and intervention stage, detailing pathways linking scorpion envenomation to clinical outcomes and public-health responses. Direct interventions include first-aid measures (e.g., wound cleaning, limb elevation, cold compresses), emergency care, management of severe systemic manifestations, and antivenom administration within healthcare systems, whose effectiveness depends on staff capacity, facility access, and antivenom availability. In resource-limited settings, traditional therapeutic practices and ethnomedicinal treatments, which are often based on medicinal plants, remain widely used, although their effectiveness and safety are variably documented (79–81). Indirect interventions complement clinical management and include scientific research, prevention strategies, and awareness activities. Prevention and awareness efforts target both healthcare workers and vulnerable populations (e.g., children, farmers, students) through community engagement and appropriate communication strategies (55, 72, 73). Scientific research in toxinology, ecology, epidemiology, and public health underpins evidence-based decision-making and integrated scorpionism control. Arrows indicate causal links, interactions, and feedbacks across components and stages, emphasizing the multi-layered, systems-based nature of scorpionism and identifying key leverage points for surveillance, prevention, clinical management, and public-health action in endemic settings.

This process generated >30 variables that informed the multi-component conceptual model presented in Figures 1, 2.

#### 2.1.8 Study selection

Records were screened by AK (title, then abstract). More than 200 documents were assessed at the abstract stage. Potentially eligible articles were then discussed with DS, and inclusion was agreed by consensus before extraction into the conceptual synthesis.

#### 2.1.9 Bibliometric component

A simple bibliometric scan was performed to identify major research domains in scorpion studies. Searches using the terms: “scorpion,” “scorpion envenomation” were run in Scopus and Web of Science.

### 2.2 Conceptual synthesis approach

This narrative and conceptual review is intended to integrate fragmented knowledge and generate hypotheses rather than to quantify effect sizes. Therefore, guided by Human Ecology Theory and socio-ecological modeling (33–35), we synthesized evidence identified through a structured literature analysis, detailed above. This synthesis, drawing on environmental, ecological, and public-health studies, grouped more than 30 determinants into six interacting components. The conceptual structure parallels DPSIR and related frameworks (27–32), but extends beyond them in several key respects. While DPSIR effectively maps drivers, pressures, state changes, impacts, and responses, it does not explicitly resolve clinical care pathways or surveillance artifacts, such as the distinction between true incidence and reported burden, nor does it explicitly integrate venom biology as a determinant of clinical severity. Similarly, One Health frameworks often focus on zoonotic transmission and cross-species pathogen dynamics, whereas in scorpionism the critical interface lies at the intersection of human exposure to arthropod hazards, environmental conditions, and health-system response capacity. The present framework therefore introduces an explicit stage transition linking upstream risk generation to downstream clinical outcomes and burden measurement, including scenarios in which improvements in surveillance increase reported incidence without reflecting true ecological change.

The model is organized into two stages: (i) a risk and vulnerability stage, encompassing climatic, ecological, venom-related, and socio-economic determinants shaping exposure; and (ii) a scorpionism occurrence stage, encompassing clinical outcomes, health-system responses, and intervention pathways. Although individual scorpion species are not explicitly listed, interspecific variation in ecology, behavior, distribution, and venom potency is embedded within the “Behavior and Ecology” and “Venom Composition” components. This species-agnostic structure ensures generalisability across endemic regions and adaptability to different taxa. The synthesized inventory of extracted variables is presented in Table 1 (Results section).

**Table 1:** Six interactive components influencing scorpion ecology and envenomation risk.

Component	Factor/Variable	Detailed evidence description	References
Physical–climatic environment	Temperature	Positive association between rising ambient temperatures and increased scorpion sting incidence; key predictor in incidence and distribution models; climate change expected to exacerbate risk.	(17–19, 38, 40)
	Thermal amplitude	Contributes to modeling scorpion diversity and spatial distribution.	(38)
	Evaporation	Higher evaporation rates correlate with increased sting incidence.	(17)
	Sunshine	Light intensity and sunshine duration are major drivers of scorpion activity and predictors in sting incidence models.	(17, 20, 40)
	Rainfall	Increased rainfall generally reduces sting incidence; low rainfall associated with higher risk.	(17, 19, 38)
	Humidity	Higher humidity correlates with reduced scorpion sting incidence.	(17, 19)
	Wind velocity/direction	Wind shows weak or negligible correlation with sting incidence.	(17)
	Climate change	High sensitivity due to sedentary nocturnal behavior and tropical/subtropical distribution; expected expansion of risk zones.	(21)
Scorpion ecology and behavior	Activity cycles	Predominantly nocturnal and sedentary; activity peaks during warm seasons.	(21)
	Habitat preferences	Distribution influenced by soil moisture, elevation, flooding risk, and temperature thresholds.	(45)
	Vegetation structure	Tree density, stem variability, and vegetation cover strongly shape scorpion assemblages.	(37, 38, 65)
	Substrate (sand texture)	Many species prefer sandy substrates due to ecomorphological adaptations.	(37)
	Foraging strategy	Sit-and-wait ambush strategy increases localized human contact.	(45)
	Reproductive seasonality	Reproduction strongly linked to climate and warm seasons.	(42, 45)
Venom composition and toxicity	Neurotoxins	Primary determinants of clinical manifestations following envenomation.	(52)
	Venom plasticity	Scorpions modulate venom composition, dosage, and sting behavior in response to ecological pressures.	(46, 47)
	Dry stings	Common defensive strategy indicating venom conservation.	(48)
Human exposure and envenomation risk	Seasonality	Sting incidence peaks in summer months (July–September).	(12, 13, 16)
	Indoor/outdoor exposure	Majority of stings occur indoors and at night.	(50)
	Geographic distribution	Risk varies regionally based on species composition and environmental factors.	(23, 39, 82)
	Vulnerable populations	Higher risk among rural populations, farmers, and children; children show higher severity.	(3, 50)
Health impacts and public health response	Clinical severity	Pain appears rapidly; severity varies by species and venom potency.	(52)
	Hospitalization patterns	Admissions peak during summer months.	(13, 15)
	Antivenom	Antivenom reduces mortality but remains debated.	(50, 53, 76)
	Surveillance	Early warning systems improve preparedness and antivenom allocation.	(83)
Socio-economic and anthropogenic factors	Land-use change	Agricultural expansion and urbanization increase scorpion–human encounters.	(55, 56, 58)
	Environmental degradation	Habitat fragmentation alters scorpion assemblage composition.	(57)
	Population exposure	Rural populations experience disproportionate envenomation risk.	(23)
	Public awareness	Education and digital communication improve prevention and response.	(77, 84)

## 3 Results

### 3.1 Bibliometric overview of scorpionism research

The complementary bibliometric search returned 4,876 documents from Web of Science Core Collection and 12,194 from Scopus database. The subset of the top 20 records ranked by database relevance was used for illustrative purposes to highlight dominant research domains. Because database “relevance” rankings are algorithm-dependent and not designed for bibliometric inference, this subset should not be interpreted as a systematic or representative sampling of the scorpionism literature. For both databases, the top 20 results (ranked by relevance) were extracted and classified by subject category. Across the retrieved records, several research areas were represented. In Scopus, the four most influential categories were Biochemistry, Genetics and Molecular Biology, Medicine, Pharmacology, Toxicology and Pharmaceutics, and Agricultural and Biological Sciences. In Web of Science, the dominant categories were Biochemistry and Molecular Biology, Pharmacology and Pharmacy, Toxicology, and Zoology.

This complementary bibliometric overview highlights a strong concentration of scorpionism research in biochemistry, toxicology, medicine, and biological sciences, with comparatively limited integration of ecological, socio-economic, and public-health perspectives (Figure 1). This imbalance further underscores the need for a unified conceptual framework capable of integrating climatic, ecological, venom-related, socio-economic, and health-system determinants, particularly if scorpionism is to be addressed alongside other neglected tropical diseases within integrated control programs. Broad disciplinary categories (e.g., engineering/physics) reflect database indexing artifacts and cross-listed journals; these represent a small minority and do not indicate major conceptual contributions to scorpionism research.

### 3.2 Components of the integrated conceptual model

**Figure 3:**
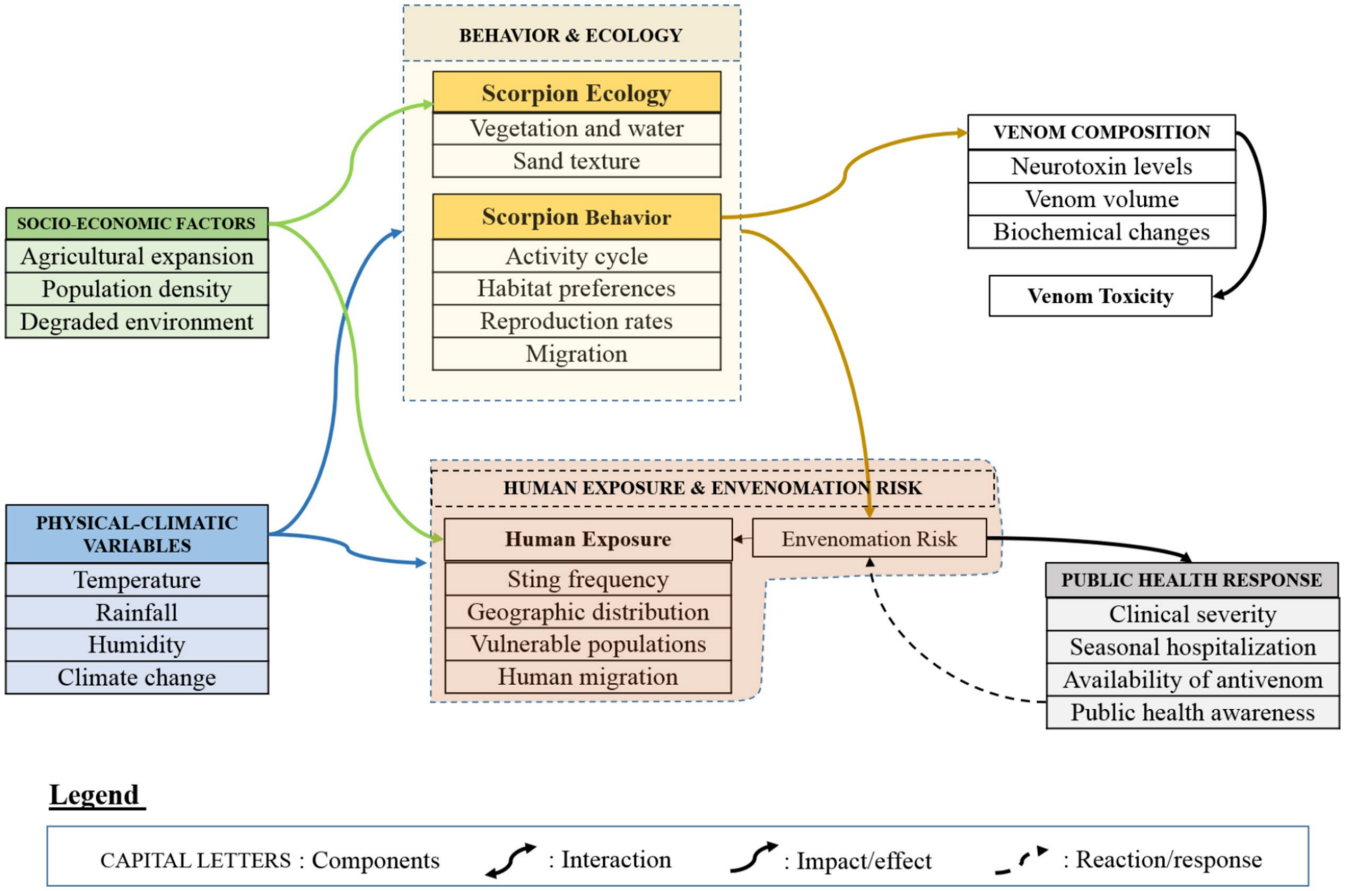
Snapshot of the most influential research areas in the field of scorpionism, collected from Scopus database **(a)** and Web of Science **(b)**. The bar plot shows the distribution of published studies across major research domains. The red dashed line highlights the four predominant areas of scientific output.

The conceptual framework is organized into six interacting components that span two stages (risk/vulnerability → occurrence/response). Each component groups determinants identified in the literature synthesis (Table 1) and is represented in Figures 2, 3.

#### 3.2.1 Physical and climatic environment

This component includes abiotic and habitat variables that characterize environmental suitability for scorpions and seasonal constraints on activity. It covers temperature and thermal amplitude, rainfall and humidity, sunshine and evaporation, wind, vegetation cover/structure, soil/substrate characteristics, water availability, and broader climatic trends such as climate change (17, 19, 20, 36–40).

#### 3.2.2 Scorpion ecology and behavior

This component captures biological and ecological traits that determine where scorpions occur and how they interact with local habitats. It includes activity cycles (e.g., nocturnality/seasonality), habitat preferences and refuge use, dispersal or migration tendencies, reproductive seasonality, foraging strategy, and other ecological attributes that shape spatial distribution and local abundance (41–45). Many medically important species exhibit nocturnal, sedentary “sit-and-wait” behavior (21).

#### 3.2.3 Venom composition and toxicity

This component encompasses venom-related determinants that influence clinical potential and severity patterns. It includes venom composition (e.g., toxin profiles, neurotoxic fractions), venom dose/volume and metering (including dry stings), and venom variability or plasticity across species, populations, and ecological contexts (46–49).

#### 3.2.4 Human exposure and envenomation risk

This component describes determinants directly related to human contact with scorpions and the probability of stings. It includes seasonality of exposure, indoor/outdoor context, geographic distribution of risk, vulnerable population groups, and behavioral or livelihood-related exposure contexts (e.g., occupation, nighttime activities, mobility/migration where relevant) (3, 12, 13, 16, 23, 50).

#### 3.2.5 Health impacts and public-health response

This component includes determinants describing the clinical spectrum of envenomation and the organization of health responses. It covers clinical severity and outcomes, healthcare utilization patterns (e.g., hospitalization), access to care and referral capacity, antivenom availability/use, and surveillance/reporting practices that shape measured burden (8, 11, 14–16, 50–54).

#### 3.2.6 Socio-economic factors

This component groups social and structural determinants that condition vulnerability, exposure contexts, and response capacity. It includes poverty and inequality, education and awareness, rurality/peri-urban settlement patterns, housing quality and peridomestic conditions, occupation and livelihoods, population density, land-use change (agricultural expansion/urbanization), and environmental degradation (22, 23, 26, 55–58).

### 3.3 Integrated synthesis of determinants of scorpion envenomation incidence

Scorpion envenomation incidence does not result from isolated determinants but from interacting socio-ecological processes linking scorpion ecology, human exposure, and health-system response, operating across environmental, socio-economic, and behavioral scales. While Section 3.2 defines the components of the conceptual framework, the present section synthesizes how these components interact along causal pathways that shape the frequency, spatial distribution, and severity of human–scorpion encounters (59, 60). Specifically, we link each determinant category to its underlying mechanisms and to the expected direction of its effect on envenomation incidence, as summarized in Table 2.

**Table 2:** Integrated synthesis of determinants of scorpion envenomation incidence, associated mechanisms, and expected direction of effects.

Determinant category	Specific determinants (examples)	Primary causal pathway	Expected effect on incidence	Mechanism (synthesis)	Key references
Climatic	Temperature (incl. seasonality), rainfall, humidity	Climate → scorpion physiology & activity → human–scorpion contact	↑ with higher temperatures; rainfall effects often non-linear/context-dependent; prolonged humidity may ↓ activity in xerophilic species	Warmer conditions increase metabolism, nocturnal activity, and movement, raising encounter probability. Rainfall can displace scorpions from shelters (short-term ↑ contact) but sustained moist conditions may suppress activity in dry-adapted species.	(60, 61, 63)
Ecological/biological	Species composition, synanthropy, population density	Ecology → medically important species presence → exposure & severity	↑ where synanthropic and highly toxic species dominate; ↑ with higher local density	Species vary in venom potency, aggressiveness, and tendency to colonize dwellings. Disturbance can favor opportunistic, medically important species, increasing both exposure and severity potential.	(62, 64, 66)
Environmental change	Land use change, agriculture, urbanization	Habitat modification → altered distribution/abundance → overlap with humans	↑ in disturbed/fragmented landscapes (context-dependent)	Habitat simplification, debris, and altered prey/refuge availability can increase scorpion persistence near settlements and raise spatial overlap between scorpions and people.	(58, 62, 65)
Housing and settlement	Housing quality, peridomestic conditions	Dwelling structure & practices → indoor colonization → sting occurrence	↑ with poor housing conditions and peridomestic refuge availability	Cracks, earthen floors, clutter, and stored materials facilitate entry and sheltering, translating ecological presence into indoor stings and increasing exposure during nighttime.	(67, 68)
Socio-economic	Poverty, occupation, education	Socio-economic vulnerability → exposure & prevention capacity	↑ in low-income populations and high-exposure occupations; education may ↓ risk via preventive behavior	Lower socio-economic status increases exposure through housing, manual labor, and limited access to protective measures. Education influences risk awareness and adoption of prevention practices.	(23, 26, 60)
Behavioral	Footwear use, sleeping habits, handling practices	Human behavior → direct exposure	Context-dependent and modifiable (protective behaviors generally ↓ incidence)	Barefoot walking, sleeping on floors, not shaking clothes/shoes, and unsafe handling increase sting probability. Simple protective behaviors (footwear, storage, shaking garments) can substantially reduce risk.	(69, 70)
Health-system and surveillance	Access to care, reporting systems, antivenom availability	Surveillance & care → recorded incidence & outcomes	↑ reported incidence with improved surveillance; true incidence unchanged (primary effect on outcomes)	Health-system capacity affects reporting completeness and timeliness of care, shaping measured burden and clinical outcomes rather than ecological occurrence of stings.	(59, 71)

“↑” indicates an expected increase in incidence; “↓” indicates a decrease; “reported” refers to changes in recorded cases due to surveillance/health-system effects.

#### 3.3.1 Climatic and environmental determinants

These climatic factors primarily influence scorpion envenomation through their effects on scorpion physiology, activity patterns, survival, and spatial distribution. Temperature is the dominant driver: higher ambient and nocturnal temperatures increase scorpion metabolic activity, foraging behavior, and movement, thereby raising the probability of human contact (61, 62). This explains the well-documented seasonality of scorpionism, with incidence peaks during warm months and heat waves in many endemic regions (60). However, extreme temperatures may reduce activity or survival in some species, indicating a non-linear relationship between temperature and sting incidence (63).

Precipitation and humidity exert more complex and context-dependent effects. In arid and semi-arid regions, rainfall events can displace scorpions from shelters, temporarily increasing encounters with humans, whereas prolonged high humidity may reduce activity in xerophilic species adapted to dry environments (63, 64). Land cover, soil type, and vegetation structure further modulate scorpion abundance by determining the availability of refuges and prey, thereby shaping the ecological baseline upon which human exposure occurs (45, 62, 65).

#### 3.3.2 Ecological and biological determinants

Species-specific traits strongly condition envenomation risk. Medically important scorpion species differ markedly in venom toxicity, aggressiveness, synanthropic behavior, and habitat preference, which directly affects both sting incidence and clinical severity (62, 64). Synanthropic species that readily colonize human dwellings, construction debris, and peridomestic environments are consistently associated with higher sting incidence and greater public health impact (66).

Scorpion population density, age structure, and reproductive cycles also influence risk, as periods of increased juvenile dispersal or mating activity may elevate encounter rates with humans (62). Importantly, ecological determinants interact with environmental change: habitat fragmentation, agricultural expansion, and urbanization often favor opportunistic species capable of thriving in disturbed landscapes, thereby increasing human exposure even when overall scorpion diversity declines (22, 56, 57, 62, 65).

#### 3.3.3 Human settlement patterns and housing conditions

Housing quality and peridomestic environmental conditions are key proximal determinants of sting occurrence. Poor-quality housing—characterized by cracks in walls, earthen floors, cluttered interiors, and lack of physical barriers—facilitates scorpion entry and indoor sheltering, substantially increasing sting risk (67, 68). Peridomestic practices such as firewood storage, accumulation of construction materials, and unmanaged waste further increase refuge availability and promote scorpion persistence near households.

Rural and peri-urban settlements often experience higher incidence due to closer proximity to natural or semi-natural habitats and limited housing infrastructure. However, rapid and unplanned urbanization can recreate similar risk conditions within cities, particularly in informal settlements, leading to sustained or increasing incidence in urban contexts (55–57, 60). Thus, housing conditions act as a key proximal determinant, translating ecological presence into actual sting events.

#### 3.3.4 Socio-economic determinants

Occupational exposure, particularly in agriculture, construction, and waste handling, increases the likelihood of encounters during peak scorpion activity periods. Education level influences risk awareness and adoption of preventive behaviors, while poverty may delay healthcare seeking, indirectly increasing morbidity and mortality following envenomation (59). Importantly, socio-economic vulnerability amplifies the effects of environmental and ecological determinants, making scorpionism both a biological and social disease.

#### 3.3.5 Behavioral determinants

Individual and household behaviors directly mediate exposure risk. Activities such as walking barefoot, sleeping on the floor, infrequently shaking clothes or shoes, and handling firewood without protection consistently increase sting probability (69, 70). Seasonal behaviors, such as sleeping outdoors during hot periods and increased nighttime activity, often coincide with periods of heightened scorpion activity, compounding exposure risk.

Preventive behaviors, when adopted (e.g., use of footwear, bed nets, improved storage practices), can significantly reduce incidence even in high-risk environments, demonstrating that behavior is a modifiable determinant capable of offsetting ecological and structural risk factors when supported by appropriate public health interventions (70).

#### 3.3.6 Health-system and surveillance determinants

While health-system factors do not influence sting occurrence per se, they strongly shape reported incidence, clinical outcomes, and perceived disease burden. Access to healthcare, availability of antivenom, and community trust in medical services determine whether stings are reported and treated promptly (59, 71). Weak surveillance systems may underestimate incidence, whereas improved reporting can generate apparent increases unrelated to true ecological change.

Public health programs that combine surveillance, community education, and environmental management have demonstrated reductions in severe outcomes and mortality, even when incidence remains high (59). Thus, health-system determinants modulate the downstream consequences of exposure and influence how scorpionism is quantified and addressed.

## 4 Discussion

Scorpionism is best understood as a socio-ecological phenomenon emerging from coupled environmental, biological, and social processes rather than from isolated risk factors (59, 60). By integrating evidence across climate science, scorpion ecology, toxinology, epidemiology, and the social determinants of health, this synthesis reframes scorpion envenomation within a systems-based architecture that captures multi-scalar drivers of exposure and disease burden. The resulting framework provides a species-agnostic model capable of accommodating regional ecological heterogeneity while maintaining conceptual generalizability across endemic settings.

### 4.1 Conceptual contribution and interpretation

The primary conceptual advance of this work lies in linking upstream environmental and socio-economic drivers with downstream clinical and surveillance outcomes within a single causal continuum. Within this structure, climatic variability, land-use change, scorpion ecological dynamics, and socio-economic vulnerability interact to generate exposure landscapes (22, 57, 62, 64), while behavioral patterns, healthcare accessibility, and surveillance performance modulate clinical outcomes and the apparent epidemiological burden (59, 71).

Crucially, the framework identifies cross-stage leverage points where targeted interventions can interrupt risk propagation. Housing quality and peridomestic environmental conditions represent proximal interfaces between ecological presence and human exposure (67, 68), while surveillance capacity and healthcare access act as downstream amplifiers or attenuators of measured disease burden (55, 59). By explicitly mapping these transition points, the framework supports a shift from single-determinant epidemiological models toward integrated systems-based prevention strategies.

More broadly, this synthesis aligns with growing evidence that environmentally mediated diseases are shaped by socio-ecological configurations rather than purely biological processes (27, 29, 32). In this context, scorpionism provides a model system for examining how environmental change, social vulnerability, and health-system capacity interact to shape disease risk under conditions of rapid climatic and land-use transformation.

### 4.2 Implications for research and public health practice

The second stage of the framework operationalizes risk generation into clinical and public-health response pathways. As illustrated in Figure 2, scorpionism outcomes are determined not only by exposure probability but also by response timeliness, treatment accessibility, and surveillance completeness (59, 71). This integrated perspective supports multi-sectoral prevention strategies that simultaneously address environmental risk management, housing improvement, behavioral prevention, clinical care, and surveillance strengthening (55, 72, 73).

From a research perspective, the framework provides a structured basis for empirical modeling of sting incidence (74), development of composite socio-ecological vulnerability indices (75), and evaluation of early-warning systems integrating climatic, ecological, and health surveillance data (28).

Operationally, the framework distinguishes determinants acting at different stages along the scorpionism pathway and can therefore guide both predictive modeling and public health decision-making. Stage 1 captures predictors of incidence risk by integrating upstream hazard and exposure processes, including climatic variability, environmental change, scorpion ecological dynamics, housing and peridomestic conditions, and human behavioral exposure. Stage 2 captures predictors of clinical severity and measured public health burden, including delays in care seeking and referral, antivenom availability and appropriate use, access to advanced supportive care (including intensive care capacity), and surveillance system performance (59, 71). A key implication is that reported incidence should be interpreted as a function of true biological incidence and surveillance completeness, such that improvements in reporting systems may increase measured burden without reflecting a true increase in sting occurrence (59). This distinction is essential for interpreting spatial and temporal trends and for separating ecological risk signals from health-system detection effects.

For empirical research and program planning, the two-stage structure can support indicator selection and help avoid conflating upstream risk generation with downstream measurement processes. Stage 1 variables are best treated as predictors of incidence risk (hazard presence and exposure opportunities) and are therefore suited to spatial risk mapping, early-warning systems, and preventive environmental and housing interventions. Stage 2 variables are best treated as predictors of severity and measured burden (timeliness and quality of care, antivenom access, referral and intensive care capacity, and surveillance completeness) and are therefore suited to improving case management, triage pathways, and surveillance strengthening (59, 71). Within this framework, increases in reported incidence may reflect improved detection and reporting even when true incidence is stable, whereas reductions in severity may result from improved access to care and effective treatment even when exposure risk remains unchanged.

Several defining features position scorpionism within the broader neglected tropical disease landscape. The burden is disproportionately concentrated among socially and geographically marginalized populations, particularly in rural and peri-urban environments where environmental exposure and structural vulnerability overlap (3, 11, 23, 25). Under-reporting remains substantial due to heterogeneous surveillance infrastructure, variable diagnostic and reporting practices, and unequal access to healthcare, leading to systematic underestimation of incidence and mortality across endemic regions (12, 15, 50). In parallel, access to antivenom and advanced supportive care remains geographically and economically inequitable, contributing to preventable morbidity and mortality despite the availability of effective treatments in some settings (53, 76, 77). While scorpionism is not formally classified as a neglected tropical disease by WHO, it aligns closely with commonly cited NTD characteristics, including concentration in marginalized populations, under-detection, and inequitable access to effective treatment and supportive care. Taken together, these characteristics support calls to strengthen the prioritization of scorpionism within NTD-oriented research and policy agendas (77, 78).

### 4.3 Limitations

This work represents a conceptual and narrative synthesis rather than a systematic review or quantitative meta-analysis. Although the literature review followed a structured search strategy, potential selection bias and uneven geographic and disciplinary representation may influence the relative weighting of determinants included in the model.

Furthermore, the framework is intentionally species-agnostic to maximize cross-regional applicability. Species-agnostic here refers to the structure of determinants and pathways; it does not assume uniform venom potency or clinical risk across taxa, but accommodates this variation within the ecology and venom components. While this enhances conceptual generalizability, it does not capture fine-scale interspecific ecological, behavioral, or venom-related variation that may influence local epidemiological patterns (41, 62). Accordingly, the framework should be interpreted as hypothesis-generating and intended to guide empirical validation rather than as a predictive or operational tool in its current form.

### 4.4 Integrated conceptual model

Scorpionism reflects the interaction of climatic, ecological, venom-related, behavioral, socio-economic, and health-system determinants operating across multiple spatial and temporal scales (59, 60). The integrated conceptual model (Figure 2) synthesizes these interactions into an end-to-end socio-ecological system linking risk generation, exposure pathways, and response capacity.

By providing a unified systems representation, the framework offers a conceptual foundation for predictive modeling, spatial risk mapping, and multi-sectoral intervention design. Framing scorpionism within a socio-ecological and neglected tropical disease perspective supports coordinated action across environmental management, clinical care, surveillance, and community-based prevention. Such integrated approaches will be increasingly critical for reducing scorpionism burden under accelerating environmental, demographic, and socio-economic change.

## 5 Conclusion

Scorpionism at the human–environment interface emerges from coupled socio-ecological processes rather than a single driver. Across the literature, three consistent insights stand out: (i) climatic variability and land-use change shape scorpion distribution and activity; (ii) envenomation risk is strongly mediated by human exposure patterns, housing conditions, and socio-economic vulnerability; and (iii) health outcomes depend on timely access to care, surveillance quality, and availability of effective treatment, including antivenom where appropriate. By integrating these findings into a unified, species-agnostic, conceptual model (risk/vulnerability → occurrence/response), this synthesis helps explain regional heterogeneity in incidence and severity and identifies leverage points for prevention and preparedness.

The overall significance of this framework is that it translates fragmented evidence into a systems structure that can guide risk mapping, early-warning development, and integrated control strategies aligned with the broader agenda of neglected tropical health conditions.

Future studies should operationalize and test this framework through: (1) empirical modeling that quantifies and compares pathway contributions (e.g., climate → ecology → exposure; socio-economic vulnerability → care-seeking delays → severity); (2) development and external validation of composite vulnerability indices using standardized indicators across endemic settings; (3) integration of species traits and venom-related variability into spatial and seasonal risk models; (4) evaluation of intervention effectiveness using quasi-experimental or implementation-science designs (e.g., housing improvement, peridomestic management, targeted education, and surveillance strengthening); and (5) improved measurement of under-reporting and access-to-care barriers to better estimate true burden and optimize antivenom allocation. Advancing these priorities will support more predictive, equity-oriented, and actionable scorpionism research and public-health decision-making under ongoing environmental and socio-economic change.

## Data Availability

The original contributions presented in the study are included in the article/supplementary material, further inquiries can be directed to the corresponding author/s.
